# Distinct oncogenic phenotypes in hematopoietic specific deletions of Trp53

**DOI:** 10.1038/s41598-023-33949-8

**Published:** 2023-05-09

**Authors:** Jayanth Kumar Palanichamy, Tiffany M. Tran, Jennifer K. King, Sol Katzman, Alexander J. Ritter, Gunjan Sharma, Christine Tso, Jorge R. Contreras, Thilini R. Fernando, Jeremy R. Sanford, Dinesh S. Rao

**Affiliations:** 1 Department of Biochemistry, All India Institute of Medical Sciences, New Delhi, India; 2 Department of Pathology and Laboratory Medicine, UCLA, Los Angeles, USA; 3 Molecular, Cellular and Integrative Physiology Graduate Program, UCLA, Los Angeles, USA; 4 Department of Medicine, UCLA, Los Angeles, USA; 5 Center for Biomolecular Science & Engineering, UCSC, Santa Cruz, USA; 6 Department of Molecular, Cell and Developmental Biology and Center for Molecular Biology of RNA, UCSC, Santa Cruz, USA; 7 Jonsson Comprehensive Cancer Center, UCLA, Los Angeles, USA; 8 Broad Stem Cell Research Center, UCLA, Los Angeles, USA; 9 Department of Pathology and Laboratory Medicine, David Geffen School of Medicine at UCLA, 650 Charles E Young Drive, Los Angeles, CA 90095 USA

**Keywords:** Cancer, Cancer models, Haematological cancer

## Abstract

Loss of function in the tumor suppressor gene TP53 is the most common alteration seen in human cancer. In mice, P53 deletion in all cells leads predominantly to the development of T-cell lymphomas, followed by B-cell lymphomas, sarcomas and teratomas. In order to dissect the role of P53 in the hematopoietic system, we generated and analyzed two different mouse models deficient for P53. A pan-hematopoietic P53 deletion mouse was created using Vav1-Cre based deletion; and a B-cell-specific deletion mouse was created using a CD19-Cre based deletion. The Vav1-P53CKO mice predominantly developed T-cell malignancies in younger mice, and myeloid malignancies in older mice. In T-cell malignancies, there was accelerated thymic cell maturation with overexpression of *Notch1* and its downstream effectors. CD19-P53CKO mice developed marginal zone expansion in the spleen, followed by marginal zone lymphoma, some of which progressed to diffuse large B-cell lymphomas. Interestingly, marginal zone and diffuse large B-cell lymphomas had a unique gene expression signature characterized by activation of the PI3K pathway, compared with wild type marginal zone or follicular cells of the spleen. This study demonstrates lineage specific P53 deletion leading to distinct phenotypes secondary to unique gene expression programs set in motion.

## Introduction

P53 was first classified as a tumor suppressor gene in 1989^1^. Since then, mutations in P53 have been identified as the most common cancer related genetic aberration^2,3^. Mutations and deletions in P53 have been observed in lung, breast, colon and many other tissue derived cancers^2,3^. Most of these mutations lead to loss of DNA binding function^4^. The main function of P53 is as a central coordinator of the response to DNA damage, in which P53 causes cell cycle arrest and subsequent apoptosis if the damage is irreversible. P53 deletion in all tissues of the mouse leads to accelerated tumorigenesis including the development of T-cell lymphomas, osteosarcomas and soft tissue sarcomas^5,6^. Given the central role of P53 in regulating the response to DNA damage, its role in T- and B-cells, which are two lineages that undergo physiological DNA damage during VDJ recombination, is of great interest.

T-cell development in the thymus proceeds through a series of ordered steps that results in the rearrangement of the T-cell receptor (TCR) and the generation of helper and cytotoxic T-cell subsets^7,8^. T-cell progenitors pass through defined stages, punctuated by stringent selection checkpoints that ensure the fidelity of the TCR. Early thymocytes prior to the beta selection checkpoint show a strong dependence on Notch signaling for their survival and continued proliferation^7,8^, but subsequently become dependent on the pre-TCR and TCR for survival signals^9^.

Studies have demonstrated the importance of the tumor suppressor P53 in regulating T-cell development in the mouse^10^. It is thought that deletion of P53 leads to an increased persistence of cells carrying aberrant V(D)J recombination products, which would otherwise be removed from the developing T-cell repertoire. P53 KO mice then develop thymic lymphoma, which consist of immature T-cells, resembling human T-acute lymphoblastic leukemia/lymphoma (T-ALL). Human T-ALL shows mutations in *NOTCH1,* resulting in constitutive activation of the Notch pathway^11^. One of the results of *NOTCH1* activation is the downregulation of P53; this is thought to be an important pathogenetic mechanism in T-ALL. In mice, constitutive activation of the *Notch1* pathway by the introduction of the transcriptionally active intracellular domain of *Notch1* (ICN) causes thymic lymphomas and leads to repression of P53 activity^12–14^.

B-cell development begins from the bone marrow hematopoietic stem cell (HSC) and passes through various developmental stages including VDJ rearrangement, culminating in the production of the IgM+ naïve B-cells in the bone marrow^7,15^. The surface marker CD19 is first expressed at the murine pro-B cell stage^15^, which corresponds roughly with the onset of VDJ recombination and definitive commitment to the B-cell lineage. Naïve B-cells leave the bone marrow to home to the spleen where they transition through T1 and T2 stages to become follicular or marginal zone B-cells. Interestingly, increased *Notch2* signaling in T2 B-cells is thought to be a key factor in their differentiation into marginal zone B-cells^16^. These latter cells are a functionally heterogeneous population of B-cells, responding to both T-independent and T-dependent antigens^17^.

The expression of *P53* and its target genes is repressed during B-cell development in the bone marrow via a *Bmi1* mediated mechanism^18^. In disease, mutations and deletions of the P53 gene have been observed in a variety of B-cell malignancies including B-cell Acute Lymphoblastic Leukemia, Chronic Lymphocytic Leukemia and Multiple Myeloma^19^.

To study the impact of cell-type specific loss of P53 on hematopoietic neoplasia, we generated two different models of *Trp53* gene deletion—a pan-hematopoietic deletion of *Trp53* and a B-cell specific deletion of *Trp53*—And characterized tumor formation, lineage, and gene expression changes in each of the models. Interestingly, we determined that hematopoietic-specific deletion of P53 develop T-cell malignancies at young ages and myeloid sarcoma/leukemia at advanced ages. Within the B-cell specific P53 deletion model, we observed a spectrum of phenotypes ranging from marginal zone expansion to marginal zone lymphoma to diffuse large/mixed B-cell lymphomas of the spleen. A mechanistic exploration identified distinctly different pathogenetic pathways within these models. This is the first study to have compared these two hematopoietic specific deletion models of *Trp53,* showing that they lead to the development of different types of malignancies. Furthermore, these tumor models can be further utilized for diagnostic and therapeutic research.

## Results

### Pan-hematopoietic P53 deficiency leads to T-cell malignancies in young mice and myeloid malignancies in older mice

To assess the role of P53 in regulating oncogenesis in the hematopoietic lineage, we crossed mice carrying a conditional allele of *P53* with exons 2-10 flanked by loxP sites with the Vav1-Cre transgenic mouse line. We obtained homozygous *P53* flox/flox mice carrying the Vav1-Cre transgene (Vav1-P53CKO) at the expected Mendelian frequencies. We confirmed genotypes through PCR for the floxed *P53* allele and Vav1-Cre transgene. Floxed *P53* mice without the Vav1-Cre transgene were controls. RT-qPCR and Western blotting of bone marrow and thymi respectively from control and Vav1-P53CKO mice showed absence of P53 expression after knockout (Supplementary Fig. 1).

We observed mice over an extended time course, up to 52 weeks of age. At 24 weeks of age, the peripheral blood in the Vav1-P53CKO mice showed a relative myeloid and T-cell hyperplasia (Supplementary Fig. 2). The percentages and absolute numbers of cells in the myeloid lineage (neutrophils, eosinophils and basophils) were all significantly increased in the Vav1-P53CKO mice, without an accompanying increase in the overall white blood cell count. Similarly, an increase in the percentage of T-cells (CD3e positive cells) was detected at the expense of B-lymphocytes (Supplementary Fig. 2). However, there was no difference in red blood cell and platelet counts. Thus, this data suggests there is a skewing of hematopoietic development towards the myeloid and T-cell lineage.

**Figure 1:**
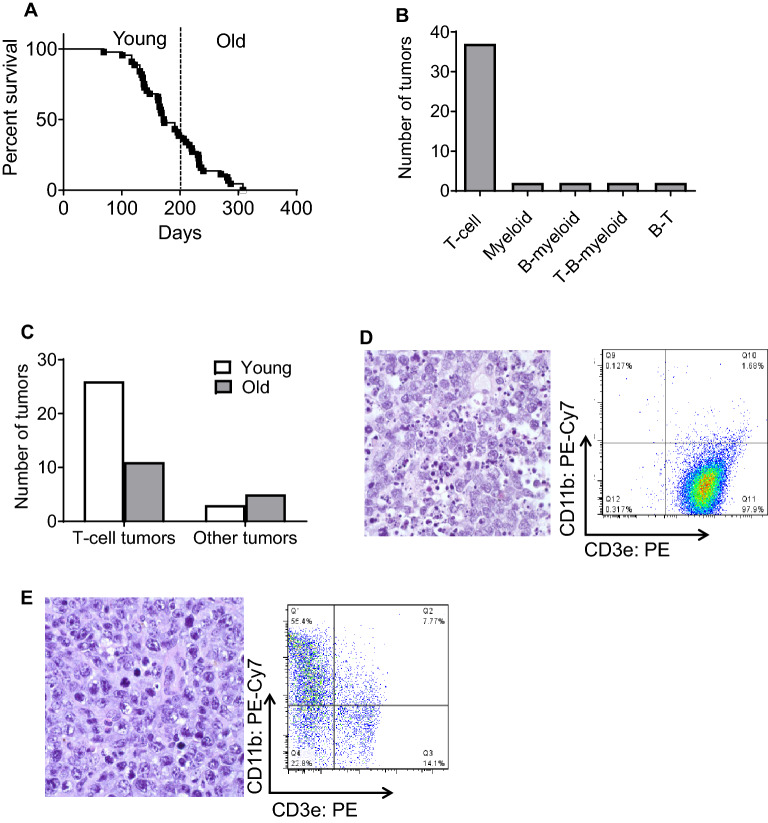
Characterization of Vav1-P53CKO malignancies and variation with age. (**A**) Survival curve of Vav1-P53CKO mice (n=45). (**B**) Classification of tumors in Vav1-P53CKO mice by surface marker expression. Markers used: T-cell (CD3e), B-cell (B220) and Myeloid (CD11b). (**C**) Stratification of tumors by age (Young < 200 days, Old > 200 days). (**D**) Microscopic image of a thymic T-cell lymphoma with the corresponding FACS analysis showing a CD3e positive lymphoma. (**E**) Histology of a splenic myeloid tumor in an old mouse and FACS confirmation of CD11b positivity in the same tumor. (**D, E**), Magnification, 1,000x; Hematoxylin & Eosin staining.

Vav1-P53CKO mice developed neoplasms specifically in the hematopoietic lineage with age (Fig. 1A). Mice with deletion of *P53* in the hematopoietic system developed hematolymphoid malignancies with a 100% penetrance by the age of 12 months. The majority of the tumors were thymic or splenic-based CD3e+T-cell malignancies (37/45 malignancies), with a significant minority comprised of myeloid and mixed lineage malignancies (Fig. 1B,C). Among these 37 T-cell lymphomas, 70% of them developed at an age of less than 200 days (26/37). Flow cytometry of the other 8 malignancies revealed mixed (T-myeloid, B-myeloid) malignancies, with a tendency towards containing a myeloid component as part of the tumor. 63% of these mixed malignancies developed in mice at an age greater than 200 days (5/8) (Fig. 1D,E). The anatomical pattern of involvement was similar with almost all hematolymphoid organs involved. Among the T-cell lymphomas, the thymus was predominantly involved in 24/37 of malignancies followed by the spleen (11/37) and other sites (2/37). Interestingly, there were no pure B-cell malignancies noted in these mice. Hence, it would appear that P53-dependent mechanisms are important in suppressing tumorigenesis in T-cell precursors in the thymus during early adulthood and in myeloid cells during old age. The shift away from T-lineage malignancies in older mice may reflect aging-related decline of lymphoid progenitors and the well-described skewing to myeloid progenitors^20^.

### T-cell malignancies in young mice predominantly display DN and DP lymphomas

**Figure 2:**
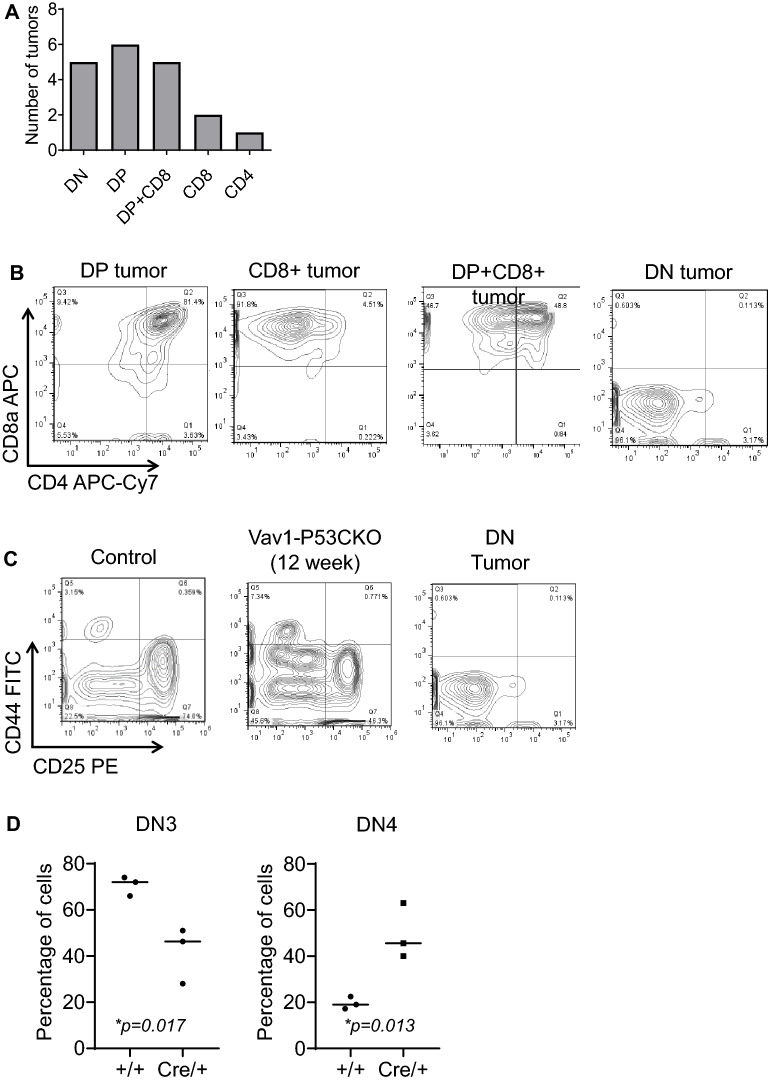
T-cell tumors from Vav1-P53CKO mice derive from thymic T-cell progenitors. (**A**) Classification of T-cell lymphomas by expression of CD4 and CD8 on the cell surface. *DP* double positive and *DN* double negative (n=19) DP+CD8+ refers to lymphomas having a mixture of DP+ cells and CD8 SP+ cells. (**B**) Representative FACS plots of different CD3e+ T-lineage lymphomas, including CD8+, Double Positive and mixed DP+/CD8+ lymphoma. (**C**) Subsetting of the T-cell precursors from a control thymus (12 weeks), pre-malignant Vav1-P53CKO thymus (12 weeks) and a Vav1-P53CKO mouse with thymic lymphoma. The scatter plots show the percentages of DN3/4 cells in the 12-week-old control and KO mice. (**D**) Quantitation of DN3 and DN4 cells in WT and pre-malignant Vav1-P53CKO thymi.

To further characterize the nature of P53-deficient oncogenesis in the thymic compartment, we performed FACS analysis of the tumors and classified them on the basis of their expression of markers that delineate their stage of development in the thymus. We found that there was a mix of tumors, including those that were double-negative (CD4-CD8-; DN), double-positive (CD4+CD8+; DP), or single positive for either CD4 or CD8 (Fig. 2A,B). Relatively speaking, the DP phenotype predominated in the P53 deficient thymic lymphomas. We also observed some tumors consisting of both DP and CD8+ cells (labeled as DP+CD8). Amongst DN lymphomas, it was most common to see lymphomas with a phenotype consistent with the DN4 stage of development (CD44- CD25-; Fig. 2C). Indeed, lymphomas with DN3 or developmentally more primitive immunophenotypes were not seen. Hence, the majority of lymphomas showed a developmental stage clustered around the beta selection checkpoint (DN4, DP). This finding may be related to the loss of a checkpoint following β-selection, where a P53- and p38 MAPK-dependent checkpoint is critical between the DN3 and DN4 stages of thymocytes development^21^**.**

To further characterize this developmental checkpoint, we analyzed hematolymphoid organs from Vav1-P53CKO mice prior to the development of overt malignancies. Thymi from 12-week old Vav1-P53CKO mice showed several alterations in T-cell development (Fig. 2C). We found the most significant change within the DN compartment. Even prior to the development of lymphomas, the mice showed a significant shift in T-cell development, with very few cells in stages DN1-3 of differentiation. Instead, the majority of the DN cells in the thymus were DN4 cells, whose numbers are normally restricted by the massive apoptosis that occurs at the DN3-DN4 beta selection checkpoint (Fig. 2D). The T-cell malignancies showed an immunophenotype corresponding to the DN4 stage and downstream developmental stages. Hence, *Trp53*-deficient T-cell neoplasms likely derive from a cell that has passed beta-selection in an abnormal fashion in Vav1-P53CKO mice.

### T-cell lymphomas show *Notch1* activation in P53 deficient mice

**Figure 3:**
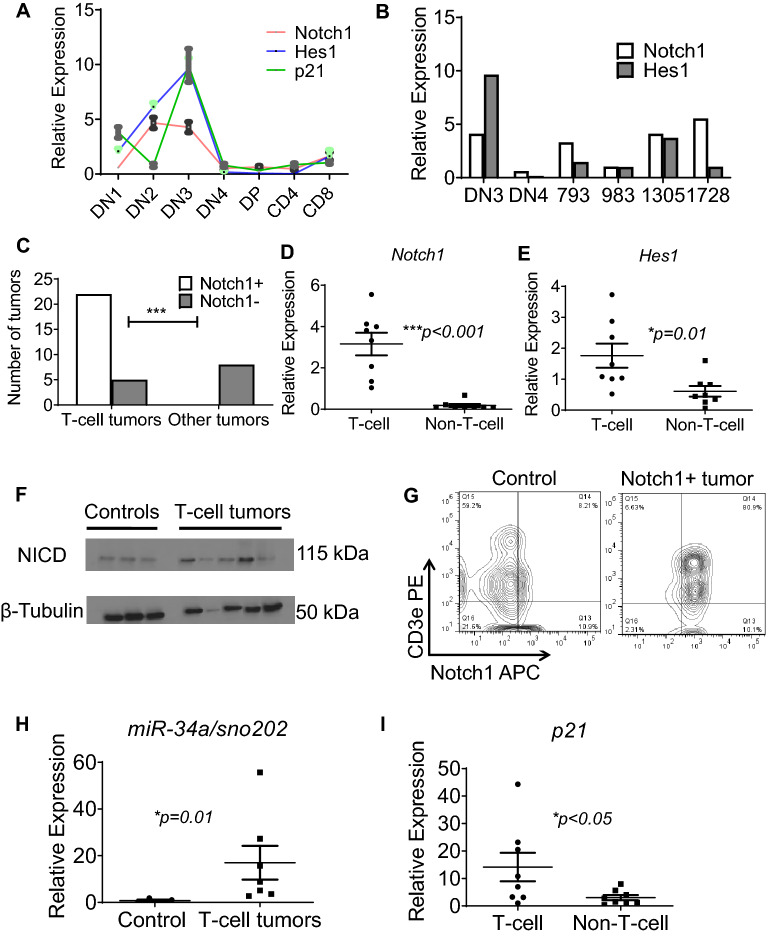
Activation of Notch1 pathway in T-cell lymphomas in Vav1-P53CKO mice. (**A**) Dynamic expression of Notch1, downstream Hes1 and p21 in murine thymic subsets *(2-way ANOVA, p=0.0024)*. (**B**) Comparison of Notch1 and Hes1 expression in the DN3 and DN4 subsets with T-cell lymphomas (numbered). (**C**) Distribution of Notch1 positivity of T-cell and other lymphomas (n=27 for T-cell lymphomas and 8 for other lymphomas); Expression of (**D**) Notch1 and (**E**) Hes1 by qPCR in T-cell (Notch1 positive) (n=8) and non-T-cell (Notch1 negative) (n=8) lymphomas. (**F**) Western Blotting for Intracellular Notch1 (NICD) from control thymi (n=3, first 3 samples from the left) and thymic lymphomas (n=5, right); Original blots presented in (**G**) Notch1 and CD3e positivity of a thymic lymphoma (**H**) Relative expression of miR-34a in control thymi (n=4) and T-cell lymphomas (n=7); sno202 was used as a reference gene (**I**) Expression of p21 in T-cell (n=8) and non T-cell lymphomas (n=8). GAPDH and L32 were used as reference genes.

*Notch1* expression has a very highly regulated trajectory during T-cell development^22,23^, and T-ALL shows activation of the *Notch1* pathway in both humans and mice. To analyze *Notch1* expression and activation during T-cell development and in P53-deficient lymphomas, we isolated murine DN thymocyte subsets and performed RT-qPCR for *Notch1, Hes1* and *p21*, which showed a dynamic expression pattern with a sharp peak in the DN3 stage (Fig. 3A). Interestingly, the T-cell lymphomas had expression of *Notch1* and *Hes1* similar to the DN3 stage rather than the DN4 stage. *p21* mRNA expression also showed a similar pattern (Fig. 3A,B). RT-qPCR on T-cell and non-T-cell lymphoma samples showed that *Notch1* mRNA levels were upregulated, concomitant with the idea that *Notch1* was being transcriptionally dysregulated in the malignant T-cells. Again, T-lineage lymphomas showed a higher level of *Notch1* activation compared to non-T-lineage malignancies (Fig. 3C,D). Furthermore, the T-lineage lymphomas also showed activation of *Hes1*, a downstream target of NOTCH1^24^ when compared to the non-T-lineage malignancies (Fig. 3E). This was confirmed by a Western blot for intracellular NOTCH1 (Fig. 3F). Given that many of the T-cell malignancies in the Vav1-P53CKO mice resemble those in human T-ALL, we stained the lymphoma cells for NOTCH1. Interestingly, we found that NOTCH1 on the cell surface was increased in lymphomas compared to normal thymi (Fig. 3G). The lymphomas with NOTCH1 positivity also showed expression of TCRβ (12/12) on their surface with no expression of TCRγ (0/12). Furthermore, there was no expression of NOTCH2/3 on these lymphomas. (Supplementary Fig. 3A,B).

The lymphomas in our mice represent a range of differentiation stages but retain NOTCH1 expression. Interestingly, the myeloid tumors did not show high levels of NOTCH1 or *Notch1* activity, consistent with the idea that *Notch1* activation inhibits myeloid development and neoplasia^25^. These findings suggest that constitutive, developmental stage-inappropriate *Notch1* upregulation occurs in P53-deficient T-cell lymphomas, and may represent a pathogenetic mechanism in these tumors.

### *Notch1* activation is multifactorial in P53 deficient mice

To explain our findings, we had hypothesized that P53-regulated miRNAs, specifically, the miR-34 family of microRNAs, may be important in the regulation of *Notch1*. *Notch1* was one of the first described direct targets of miR-34 miRNAs, which are transcriptionally induced by P53^26^. In contrast to what we had expected, miR-34a and p21 expression was upregulated in lymphomas compared to normal thymic tissue (Fig. 3H,I). This led us to examine alternate explanations for the upregulation of *Notch1* in P53-deficient lymphomas. Centrally, we hypothesized that in wild-type T-lineage cells, P53 expression normally leads to the expression of certain factors that downregulate *Notch1*. *Notch1* expression is tightly controlled during development by multiple pathways including NUMB, MDM2 and GSK3β^27–30^. In fact, we found increased MDM2, increased active GSK3β and decreased NUMB levels, all of which would be expected to contribute to increased *Notch1* activity (Supplementary Fig. 4A). In addition, we found that the majority of the lymphomas showed a dramatic change in the RNA isoform of *Ikaros*, which is known to be a repressor of *Notch1* target genes^31,32^. Indeed, this isoform of *Ikaros* was seen in the DN3 stage of differentiation of T-cells and is downregulated subsequently (Supplementary Fig. 4B–D). Hence, the mechanisms whereby P53 deficiency acts to increase NOTCH1 levels likely involves several pathways, including MDM2, GSK3β, NUMB and IKAROS, all of which are central in oncogenesis and development.

### B-cell specific deletion of P53 leads to massive splenomegaly and indolent disease

**Figure 4:**
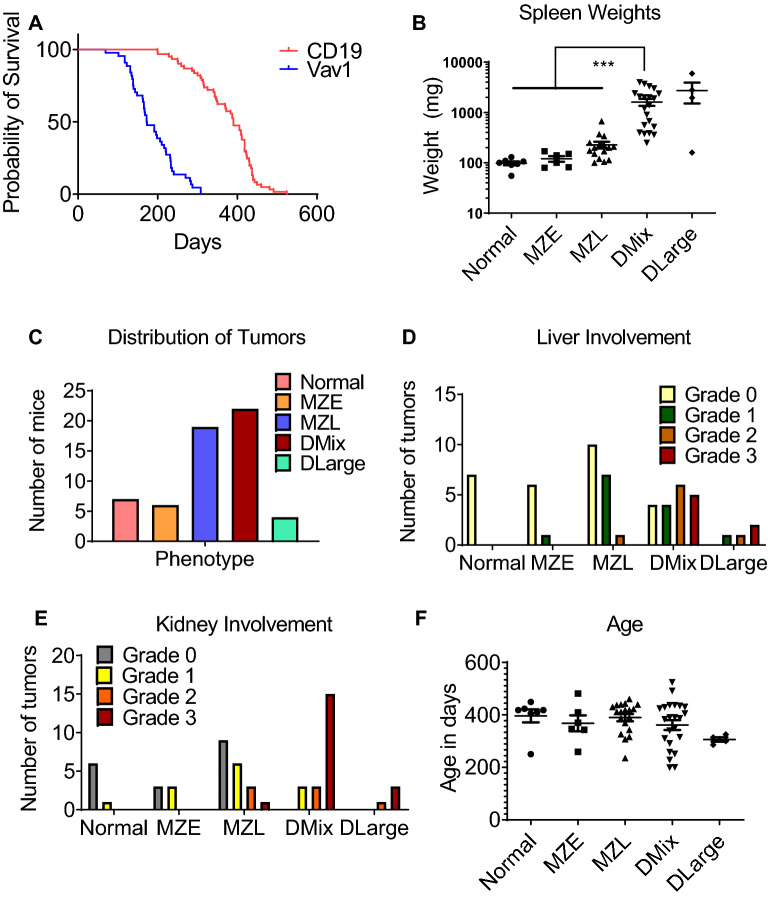
Characterization of CD19-P53CKO mice and lymphomas. (**A**) Survival curves of Vav1-P53CKO (n=45) and CD19-P53CKO (n=54) mice *(Log-rank (Mantel-Cox) test, p<0.0001)*. (**B**) Spleen weights of different groups of mice (normal n=7; marginal zone expansion (MZE) n=6; marginal zone lymphoma (MZL) n=16; diffuse mixed lymphoma (DMix) n=21; diffuse large cell lymphoma (DLarge) n=4). Significantly higher weight seen in DMix and DLarge compared to other groups (One way ANOVA ***p <0.00001). (**C**) Distribution of lymphomas in the CD19-P53CKO mice (**D**) Quantification of Liver and (**E**) Kidney involvement (**F**) Age distribution of these lymphomas.

To investigate the function of P53 in B-cells, we crossed the CD19-Cre (B6.129P2(C)-CD19 Cre) with the floxed P53 mouse to create a B-cell specific knockout model of *P53* (CD19-P53CKO). The CD19-Cre is a knock in model with *Cre* replacing the endogenous *CD19* protein coding gene. We utilized mice homozygous for the floxed P53 allele and heterozygous for the CD19-Cre allele for our studies. To confirm lineage specific *P53* deletion, we used B220 antibody coated beads and MACS to separate B-cells from the spleen. RT-qPCR showed no *P53* expression in the B220+ fraction and *P53* expression in the B220- fraction confirming B-cell specific deletion. Western blotting of spleens from the CD19-P53CKO mice also confirmed the lack of *P53* expression (Supplementary Fig. 5). The CD19-P53CKO mice (n = 54) were followed up to the age of 2 years. Median survival was 390 days (Fig. 4A). The majority of mice (47/54) developed splenomegaly in an age-dependent manner, while 7/54 did not show splenomegaly at the experimental endpoint. 26/47 had severe splenomegaly with spleens weighing >300 mg, with or without involvement of other lymph nodes, such as the neck, mesenteric and inguinal nodes. 14/26 had spleen weights greater than 1 gram with the largest spleen weighing 5.96 grams (Fig. 4B). Thus, B-cell specific deletion of *P53* results in a latent, indolent disease, and occasionally massive splenomegaly.

### Histological and flow cytometric analysis of splenic tumors show variable phenotypes

**Figure 5:**
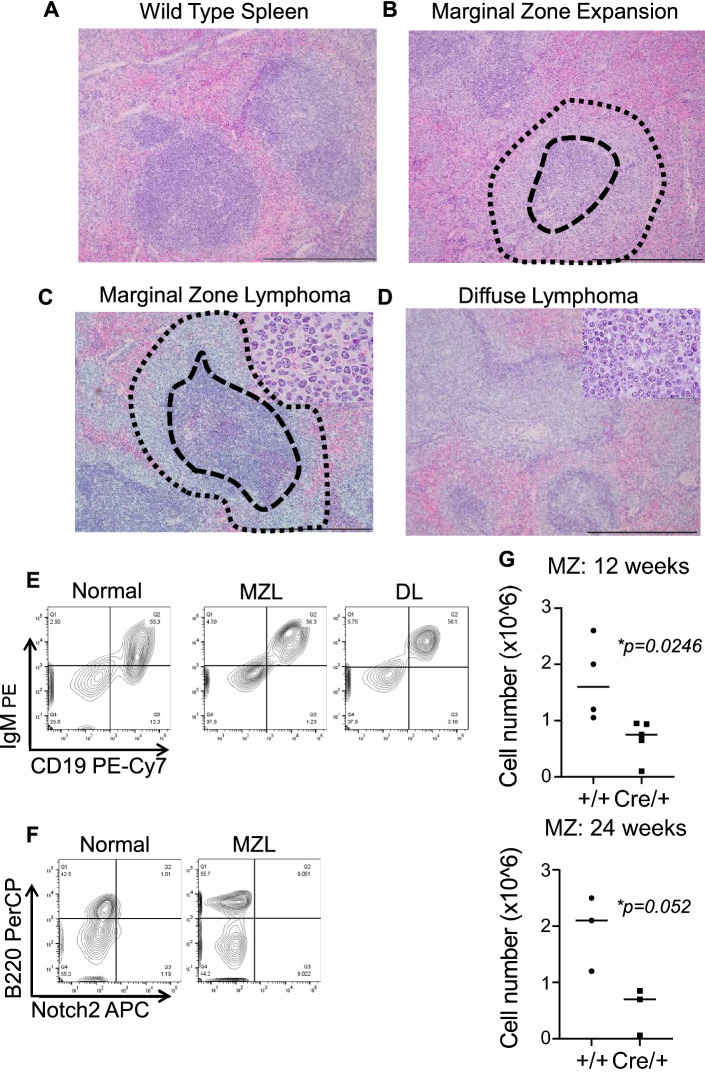
Histologic and flow cytometric characterization of CD19-P53-CKO lymphomas. (**A**–**D**) Histologic appearance of normal spleen (wild type mice) (**A**), marginal zone expansion (**B**), marginal zone lymphoma (**C**) and diffuse lymphoma (**D**) at 100X magnification; insets depicting the morphology of the lymphoma cells are shown at 1000X magnification.  Scale bars, 400 µm. The region between the dashed and dotted line contains the marginal zone, while the region within the dashed line constitutes the follicular zone.  Flow cytometric analysis shows CD19+ IgM+ lymphoma cells (**E**) which are negative for Notch2 (**F**). (**G**) Flow cytometric analysis of 12-week and 24-week WT and KO mice spleens showing significantly reduced marginal zone cell numbers.

Upon histologic examination, splenomegaly was characterized by marginal zone expansion (6/54), low-grade marginal zone lymphoma (16/54), and diffuse lymphoma (25/54) (Fig. 4C). Diffuse lymphoma showed a histology characterized by involvement of both the white pulp and the red pulp, as opposed to predominant localization to marginal zones within the white pulp (Fig. 5A–D). The diffuse splenic lymphomas were further classified into diffuse mixed B-cell (mixture of small and large cells: 21/54) and diffuse large B-cell lymphomas (4/54) (Figs. 4C, 5A–D). All mice displaying splenomegaly greater than 1 gram (14/26) showed diffuse lymphoma on histological analysis, suggesting a correlation between the histologic subtype and increased splenic size.

The disease was confined to the spleen in the case of lower-grade histology while higher grade histology correlated with involvement of the lymph nodes, thymus, liver and kidney. Liver and kidney infiltrates were graded from 0 (no involvement) to 3 (extensive involvement) by microscopy of H&E stained sections. There was a correlative increase in higher grades of liver (*p=0.001 Chi-square test*) and kidney (*p<0.0001)* infiltrates with higher grade splenic disease. In fact, most of the Grade 3 and 4 infiltrates were seen only in mice with diffuse splenic lymphoma *(p=0.007* *for* *MZL* vs. *Diffuse Mixed Cell Liver Infiltrates and p<0.0001 for Kidney Infiltrates, Chi-square test).* Spleen weights followed a similar trend with the highest weights seen in the diffuse type of splenic lymphoma. However, there was no age specific difference between the subtypes (Fig. 4D–F).

Interestingly, progression of the indolent splenic marginal zone lymphoma (SMZL) to the more aggressive diffuse large B-cell lymphoma (DLBCL) has previously been reported in humans. Flow cytometric analysis of the spleen showed the presence of B220+CD19+IgM+ lymphoma cells along with the normally present T-cells. Where involved, the nodes were also B220+ IgM+ lymphomas. Bone marrow involvement was uncommon with 7/47 showing increased B220+ cells in the marrow (Fig. 5E). Therefore, B-cell specific *P53* deletion in mice display progressive development of diffuse splenic lymphoma, similar to human disease.

### Marginal zone B-cells are not increased in B-cell P53-deficient mice prior to lymphoma development

NOTCH2 appears to be a critical regulator of normal marginal zone development^33^, and activating mutations in *Notch2* have been observed in 20% of human splenic marginal zone lymphomas^16,34^. Given these findings, we queried overexpression of NOTCH2 in CD19-P53CKO lymphoma, finding no significant expression (Fig. 5F). 29/31 of analyzed lymphomas were IgM positive by flowcytometry *(Data not shown)*. BCL6 expression was also present in these tumors indicating the presence of somatic hypermutation (Supplementary Fig. 5).

This led us to analyze the immunophenotypic properties of the pre-lymphomatous B-cells in CD19-P53CKO mice. We used flow cytometry-based analysis of the splenic fractions of floxed *P53* and CD19-P53CKO mice. Using CD21, CD23, CD93, B220 and IgM surface markers, we characterized the follicular, marginal zones, and T1/T2 B-cell compartments in the spleens of two groups of mice at 12 weeks and 24 weeks (n=4/group). In younger as well as older mice, there was no difference in the total number of follicular, T1 and T2 cells in the spleen of floxed *P53* and CD19-P53CKO mice. However, there was a significant decrease in the number of marginal zone cells in CD19-P53CKO spleens. This suggested that the marginal zone expansion does not arise from classical marginal zone cells, as defined by immunophenotype (Fig. 5G). Rather, it suggests that the neoplastic B-cells may derive from a different stage of B-cell development, which then colonize marginal zones in the spleen.

### The transcriptome of CD19-P53CKO lymphoma differs from normal B-cell subsets

To further query the differences between B-cell lymphoma and normal B-cell subsets, we performed transcriptome profiling of sorted follicular and marginal zone B-cells from floxed *P53* and CD19-P53CKO mice at 12 weeks and 24 weeks (n=3 from each group), compared with diffuse lymphoma cells from 5 CD19-P53CKO mice. Surprisingly, there was an extremely similar transcriptome with only ~10 genes differentially expressed at both 12 and 24 weeks between the floxed *P53* and the CD19-P53CKO fractions (follicular and marginal zone). Interestingly, the PCA plot also showed a clear separation of the follicular B-cells, marginal zone B-cells and the lymphomas into three distinct clusters irrespective of age and P53 status (Supplementary Fig. 6). However, when these fractions were compared against the 5 lymphomas, there were >10,000 genes significantly differentially expressed, demonstrating a unique transcriptome which developed during malignant transformation (Supplementary Table 2)*.*

**Figure 6:**
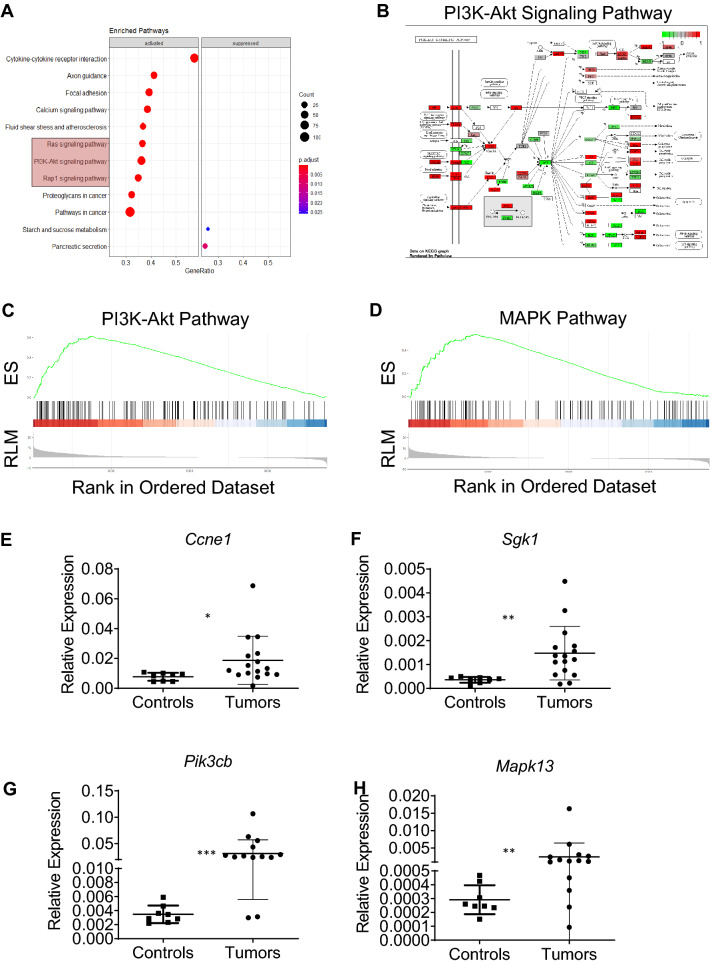
Transcriptomic analysis of CD19-P53CKO lymphomas. (**A**–**D**) GSEA analysis of differentially expressed genes in CD19-P53CKO lymphomas shows an enrichment for PI3K, Rap1 and MAPK pathways (*ES* enrichment score; *RLM* ranked log metric). (**E**–**H**) Validation of PI3K and MAPK targets by qPCR in lymphomas (n=13) and controls (n=8). Controls included sorted marginal zone cells from 12-week-old WT or CD19-P53CKO mice.

To determine the signaling pathways enriched within this dataset, we completed pathway analysis of these differentially expressed genes using Gene Set Enrichment Analysis (GSEA). Within the KEGG pathways, we found the PI3K, RAP1 and MAPK signaling pathways, which are associated with cellular proliferation, to be significantly enriched (Fig. 6A–D). We identified candidate genes from these pathways and did a validation analysis on 13 lymphoma samples by RT-qPCR. Fractions from the control mice (follicular/marginal zone B-cells) were used as controls. Since there was no significant difference between the gene expression between the fractions, they are presented as a single group. There was a significant overexpression of the *Ccne1*, *Sgk1*, *Mapk13* and *Pik3cb* genes by qPCR, validating the role of these enriched pathways after P53 knockout (Fig. 6E–H). Therefore, frank lymphomagenesis in the context of P53 deficiency leads to an increase of multiple genes involved in key cellular signaling pathways, including the PI3K/MAPK pathway, for cellular proliferation.

## Discussion

### Complete hematopoietic knockout of P53

*P53* is one of the most important tumor suppressor genes characterized. It is the most common gene to be mutated in human cancers (50% of tumors). Mutation leads to loss of DNA binding which prevents the normal downstream functioning of wild type P53 which include the activation of cell cycle arrest and apoptotic pathways during cellular stress^2,3^. In mouse models where *P53* is deleted in the whole animal, there is accelerated oncogenesis similar to the human disease Li Fraumeni syndrome, where functional P53 is lost^5,35^. In *P53-/-* mice, a preponderance of thymic lymphomas has been reported. Most of these tumors develop by 6 months of age and express both CD4 and CD8 markers on their surface (Double Positive phenotype). T-cells from *P53–/–* mice show increased genomic instability, which might contribute to the development of tumors^36^. A block in T-cell development in *P53–/–* mice by inactivating the TCRβ enhancer lead to persistent RAG endonuclease activity and accelerated lymphomagenesis^37^. Interestingly, crossing these *P53–/–* mice with *Rag1/2*-deficient mice did not inhibit lymphomagenesis, demonstrating that lymphoma development was independent of VDJ recombination, and may be related to accelerated cycling through thymic developmental stages^38,39^.

T-cell development in the thymus proceeds through a series of ordered steps that result in the rearrangement of the T-cell receptor and the generation of helper and cytotoxic T-cell subsets^7,8^. These T-cell progenitor cells then pass through several immunophenotypically defined stages, punctuated by stringent selection checkpoints that ensure the fidelity of the TCR. Early thymocytes, at stages preceding the beta selection checkpoint, show a strong dependence on *Notch1* signaling for their survival and continued proliferation^7,8^, but subsequently become dependent on the pre-TCR and TCR for survival signals^7–9^. Here, we created a hematopoietic-specific knockout of *P53* and found that mice deficient for this gene develop T-cell malignancies at young ages and myeloid sarcoma/leukemia at advanced ages. Immunophenotypically, the T-cell malignancies were all derived from stages corresponding with cells that have passed (or bypassed) the beta selection checkpoint. Interestingly, these malignancies showed a persistent elevation in the expression levels and activation of *Notch1* signaling via multiple mechanisms.

Human B-cell/T-cell malignancies with P53 inactivation have been shown to accumulate Immunoglobin/TCR locus translocations respectively. HSC specific (Vav1-P53CKO) or Thymocyte specific (Lck-P53CKO) deletion of P53 in mice have been shown to lead to the development of TCRβ+ DP or DN thymic lymphomas with clonal translocations^11^. We observed Vav1-P53CKO mice for an extended time period with detailed analyses of thymic subsets. We observed that the younger Vav1-P53CKO mice predominantly developed T-cell lymphomas and older mice tended to show an increased incidence of myeloid or mixed lineage tumors. The T-cell lymphomas were a mixture of DP, DN and SP (CD4+/CD8+) lymphomas. At 12 weeks, there was accelerated cycling of Vav1-P53CKO thymocytes from the DN3 to DN4 stage. DN3 is the first checkpoint in T-cell development (β-selection) where a properly rearranged pre-TCR is necessary to transmit signals to continue development^9^. This DN3 checkpoint is controlled by the p38 MAP Kinase which activates P53 which subsequently causes G2/M arrest^40^. This is speculated to be critical to allow DNA repair produced by V(D)J-mediated double stranded breaks and prevent retention of abnormal translocations. In the absence of P53, we observe a loss of this checkpoint, and thymocytes with the DN3 genotype appear to continue abnormal (and unchecked) development to subsequent stages. In the thymic lymphomas, this discordance is evident in the persistent expression of *Notch1, Hes1* and *p21*, as well as the persistent expression of *Ikaros* isoforms normally seen in the DN3 stage.

Many epigenetic and other mechanisms serve to tightly regulate *Notch1* signaling: the expansion of T-cell progenitors prior to beta selection is entirely *Notch1* dependent; thereafter, it is *Notch1*-independent. Activating *Notch1* mutations have been observed in ~30% of mouse T-cell lymphomas^12^. *Notch1* and its downstream *Hes1* levels increase steadily up to the DN3 stage and fall thereafter, most likely secondary to an increase in the *Ikaros* (IKZF1) protein levels, which represses *Notch1*. Following beta selection, *Notch1* expression is dramatically downregulated, presumably via proteolytic degradation, and the expression of its target genes also becomes downregulated. The T-cell lymphomas overexpress *Notch1* and the mechanism behind this appears to be multifactorial. NUMB is one of the factors which forms a complex with P53 and MDM2 and inhibits NOTCH1 activity in breast cancers^41^. This inhibition is by accelerated endocytic sorting promoting NOTCH1 degradation in an ITCH1 dependent manner^42^. We demonstrate that there is decreased NUMB and increased MDM2 expression in NOTCH1 overexpressing lymphomas. Active GSK-3β increases the levels of NOTCH1 protein and its active intracellular domain, NICD^30^. In the T-cell lymphomas in our model, there is an increase in Total GSK-3β and a decrease in its inactive phosphorylated form. Overall, we demonstrate multifactorial mechanisms leading to *Notch1* signaling activation and subsequent acceleration through the T-cell developmental stages in this model of pan-hematopoietic P53 deletion. Thus, the Vav1-P53CKO mouse may be used as a pre-clinical model to study thymic T-cell lymphomas. However, it does not entirely mimic human tumors which are thought to evolve from a clone of cells that accumulate mutations in a specific order, based on survival advantage and feedback from their microenvironment as compared to the pan-hematopoietic/pan B-cell specific KO of P53 in the mouse models. This is a limitation of the model which also applies to the CD19-P53CKO model as well.

### B-cell specific P53 knockout

Germline deletion of *P53* in mice can rarely lead to the development of lymphomas of B-cell origin. However, marginal zone hyperplasia and indolent lymphomas have previously been described in mice where P53 was deleted in all cells^43^. These lymphomas expressed IgM and had clonal IgH gene rearrangements. Another research group created and characterized a CD21-Cre based model of P53 knockout. CD21 is a mature B-cell marker and is expressed much later during the B-cell ontogeny than CD19. These mice also developed marginal zone lymphomas which harbor recurrent translocations between the Immunoglobulin loci and critical oncogenes including *C-MYC*, *BCL2* and *BCL6*^44^. Another group attempted to create a reversible model of P53 knockout by introducing a stop codon in the first exon of P53 which would then be excised by a Cre-recombinase leading to tissue specific expression of P53^45^. However, these P53 knockout mice unexpectedly retained P53 expression in all tissues except B-lymphocytes, resulting in a model of B-cell specific P53 deletion. 11/29 of these mice also developed splenic marginal zone lymphoma, similar to both the germline deleted mice and the CD21-driven conditional deletion. We created a pro-B cell specific CD19+ B-cell specific deletion of P53 using a CD19-Cre crossed to the floxed P53 mouse model. We observed the development of indolent marginal zone lymphomas at an average age of ~1 year. There was a gradual progression from marginal zone expansion to marginal zone lymphoma, which progressed to diffuse mixed cell or large cell lymphoma. This histologic progression was correlated to a corresponding increase in spleen weights and worsening of liver and kidney infiltrates. This is in line with both the previously published studies.

In the spleen, the marginal zone (MZ) forms the boundary between the circulation and the white pulp. MZ B-cells were suggested to be similar to the B1 subtype of B cells, and speculated to play a role in defence against blood-borne pathogens in a T-cell independent manner. They have a lower activation threshold than follicular B-cells and help in the rapid production of IgM+ B-cells. Recent research also suggests that MZ B-cells can also undergo T-cell dependent stimulation and antibody production. The development of MZ B-cells is dependent on the activation of B-cell receptor (BCR), BAFF receptor and the NOTCH2 receptors^16,46^. In the lymphomas which develop in CD19-P53CKO mice, there is an initial marginal zone expansion followed by a marginal zone lymphoma which progresses to the diffuse lymphoma. However, these lymphomas show no NOTCH2 or CD21 expression which are characteristic features of the normal MZ B-cell. Transcriptomic analysis of these lymphomas along with follicular and marginal zone B-cell subsets from the spleens of WT/P53 CKO spleens followed by GSEA analysis identified enrichment of numerous pro-proliferative pathways including the PI3K, MAP Kinase, RAP and RAS signaling pathways. Validation of few genes in these pathways confirmed this overexpression in a larger cohort of lymphomas. Interestingly, the BCR pathway also activates the PI3K pathway. A double knockout mouse model (PTEN+/- and LKB+/) with constitutive activation of the PI3K-mTOR pathway was reported to develop massive splenomegaly and marginal zone lymphoma which also involved lymph nodes^47^. These data appear to phenocopy the phenotype which we have seen in B-cell-specific P53-deficient mice.

In humans, splenic marginal zone lymphomas (SMZL) are indolent lymphomas characterized by mutations in KLF2, NOTCH2 and P53 genes. Transcriptomic analysis followed by GSEA analysis of human SMZLs has previously identified upregulation of the NF-κβ and BCR signaling pathways^34,48,49^. Very few studies are available about the pathways deregulated in SMZL and no proper mouse models are available for the same^50,51^. Our CD19-P53CKO model may help model human SMZL and serve as a preclinical model for carrying out SMZL research in the future.

## Materials and methods

### Mice

Flox/flox P53(B6.129P2-TrP53tm1Brn/J), Vav1-Cre (B6.Cg-Tg(Vav1-Cre)A2Kio/J) and CD19-Cre mice (B6.129P2(C)-Cd19tm1(Cre) Cgn/J) were obtained from Jackson laboratories. The Vav1-Cre/CD19-Cre mice were interbred and backcrossed onto flox/flox P53 mice to obtain flox/flox P53 mice with presence (test) or absence (control) of the Vav1-Cre/CD19-Cre transgene. Genotyping for P53 as well as Vav1-Cre/CD19-Cre confirmed the genotype. Genotyping was performed as per the protocols provided by Jackson Laboratories.

All mice were housed under pathogen free conditions at the University of California, Los Angeles. At UCLA, all mouse experimental procedures are approved by the UCLA Institutional Animal Care and Use Committee, known as the Chancellor’s Animal Research Committee (ARC). This study was scrutinized and approved by the same.

The reporting in the manuscript follows the recommendations in the ARRIVE guidelines. For retroorbital bleeds, isoflurane anesthesia was used. Mice were sacrificed at the experimental endpoint or upon the development of any pre-moribund conditions, in accordance with policies regulating animal research. In this study, euthanasia was performed by CO_2_ asphyxiation method as recommended by the ARC guidelines.

### Flow cytometry

Blood, bone marrow, spleen, thymic tissue and lymphomas were collected from the mice under sterile conditions. Single cell suspensions were lysed in red blood cell lysis buffer. A panel of fluorochrome conjugated antibodies were used for staining (List of antibodies and Schematic in Supplementary Table 1). Flow cytometry was performed on at the UCLA Flow Core Facility and analysis performed using FlowJo software as previously described^52^.

### Histopathology

Organs were collected after necropsy and fixed in 10% neutral buffered formalin. These were then embedded in paraffin, processed for hematoxylin and eosin staining by the Translational Pathology Core Laboratory at UCLA. Histopathologic analysis was performed by a board-certified hematopathologist (DSR).

### FACS sorting of splenic subsets

The follicular and marginal zone subsets from young and old wild type and CD19-P53CKO mice were sorted on a FACS Aria III using markers and protocols already standardized in the lab^53^.

### Statistical analyses

Figures are graphed as mean with the standard deviation of the mean (SD) for continuous numerical data. Bar graphs are employed to show dichotomized or ordinal-type histopathologic data. Student’s *t* test, Fisher’s exact test, Chi square test, and Kaplan-Meier survival analysis were performed using GraphPad Prism software, applied to each experiment as described in the figure legends. Additional methods are mentioned in the Supplementary methods file.

## Supplementary Information

Supplementary Figures.

Supplementary Information.

Supplementary Table 2.

## Data Availability

The RNA-Seq libraries from the various subsets and tumors were sequenced on an Illumina HiSeq 2000 (single-end 50bp). Raw sequence files were obtained using Illumina’s proprietary software and were deposited in the National Library of Medicine/ National Center for Biotechnology Information (NCBI) as a BioProject under Accession # PRJNA888125.
