# Supplementary material for: Distinct oncogenic phenotypes in hematopoietic specific deletions of Trp53
Source: Sci Rep. 2023 May 9;13:7490. doi: 10.1038/s41598-023-33949-8 (PMC10169790; doi:10.1038/s41598-023-33949-8)
Supplement: Supplementary file 1 — Supplementary Figures. [file 41598_2023_33949_MOESM1_ESM.pdf]

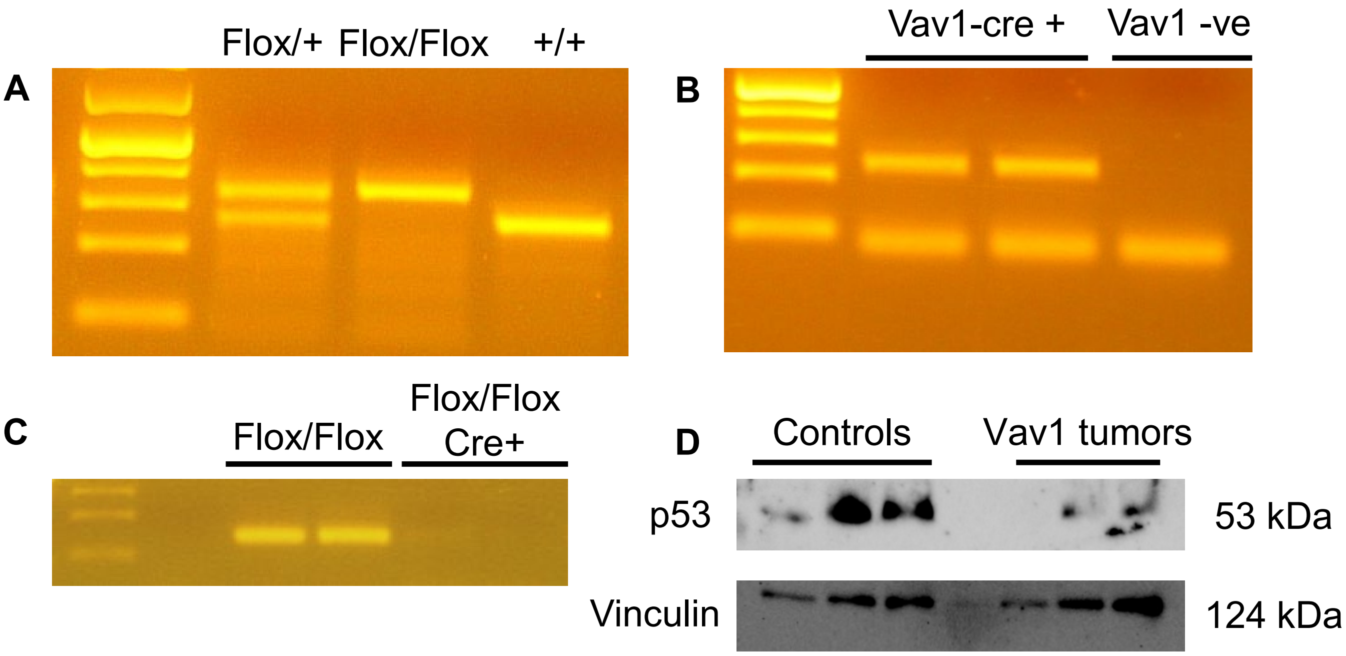

Supplementary Figure 1: A) Genotyping for the floxed p53 gene. The floxed gene gives a 370 bp band whereas the wild type p53 gene gives a 290 bp band which is evident from the banding pattern in the picture. B) Genotyping for the Vav1-cre transgene shows two positive samples (236 bp band) and a negative sample C) RT-PCR for P53 expression from the bone marrow of Flox/flox (Control) or Flox/Flox, Vav1-Cre+ Bone marrow reveals complete lack of P53 expression D) Western blot for P53 in control thymi (Lanes 1-3) and thymic tumors from the Vav1-Cre mice (Lanes 5-7) shows very little P53 protein expression in the tumors. Vinculin was used as a loading control; Original blots presented in Supplementary Figure 7

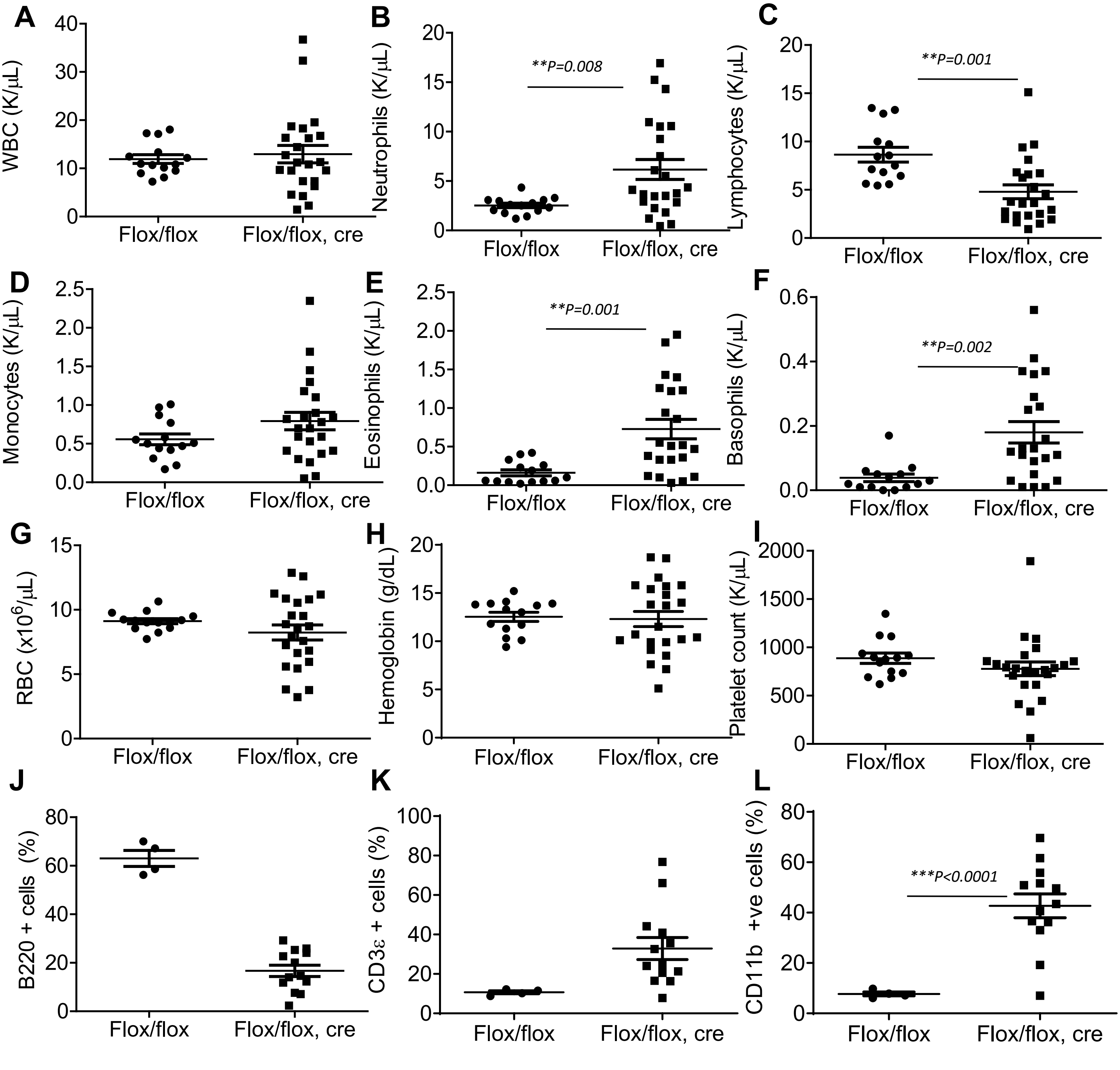

Supplementary Figure 2: Peripheral blood analyses in Vav1-p53CKO mice. The peripheral blood was collected from control (n=14) and Vav1-p53CKO (n=22) animals at 24 weeks A) Total White blood cell (WBC) counts. Absolute numbers of B) Neutrophils C) Lymphocytes D) Monocytes E) Eosinophils F) Basophils G) RBCs H) Hb levels and I) Platelet counts. Also shown are percentages of B220+ B-cells (J) and CD3e+ T-cells (K) by FACS.

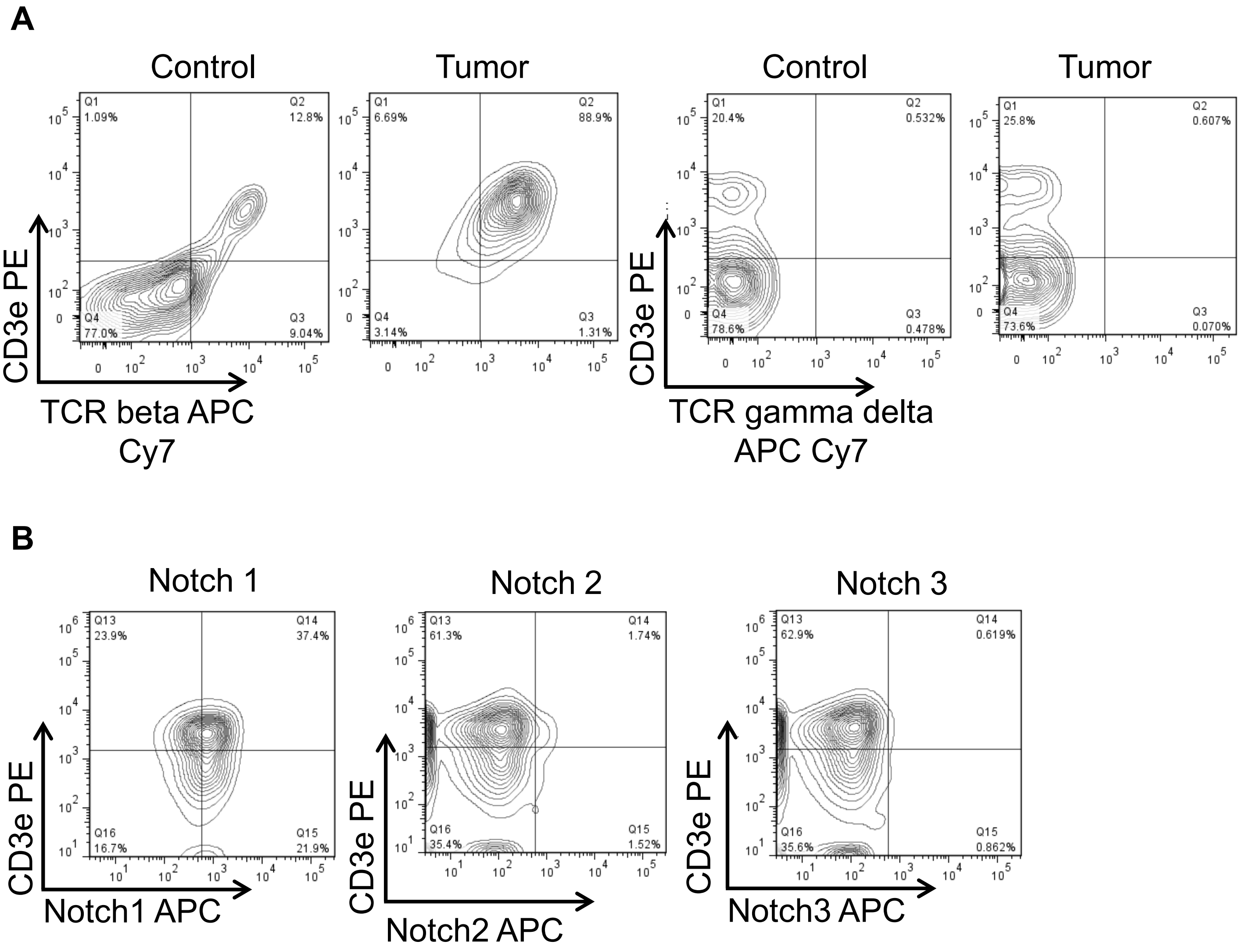

Supplementary Figure 3: Thymic tumors express TCRβ (A) and Notch1 but not TCRγ and Notch2/3 (B)

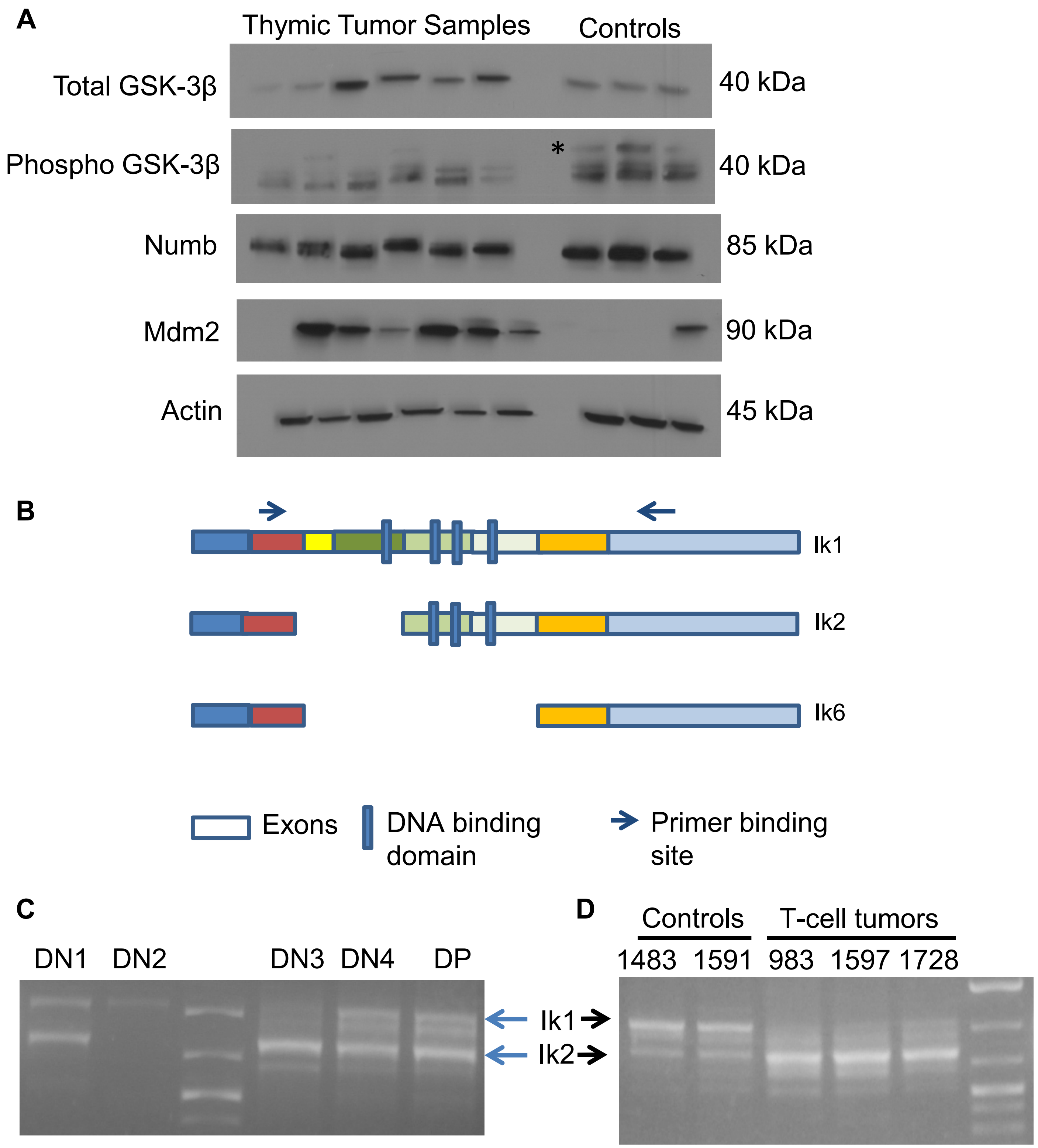

Supplementary Figure 4. Mechanisms of Notch1 pathway activation in Vav1-p53CKO mice: A) Western blot of the mentioned proteins in 6 Notch1 positive thymic tumors and 3 control thymi (48-, 24- and 12-week-old thymi, from left to right; * refers to the correct Phospho GSK-3β band) B)Schematic of the exon structures of the various Ikaros transcripts and the locations of the primers used. C) Dynamic expression pattern of the Ikaros isoforms (1 and 2) in different subsets of the thymocytes by RT-PCR D) Comparison of Ikaros transcript expression in control thymi and thymic T-cell tumors by RT-PCR; Original blots presented in Supplementary Figures 8-10

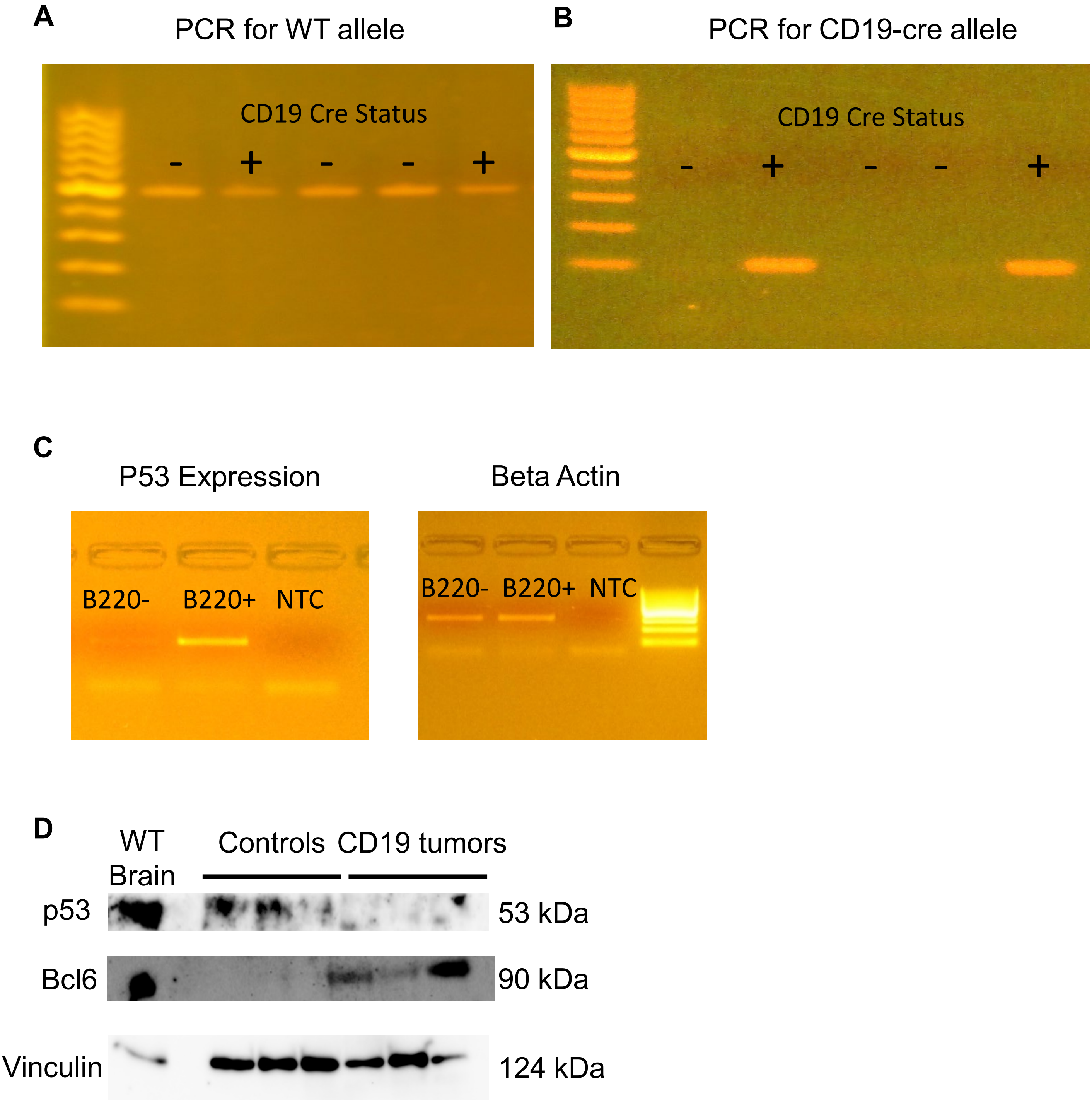

Supplementary Figure 5: Genotyping for the CD19-cre transgene. The wild type allele gives a 477 bp band (A) whereas the CD19-cre allele gives a 100 bp band (B) C) RT-PCR for P53 expression after MACS sorting for B220+ cells from a CD19-P53-CKO spleen from the flowthrough (B220- cells) and eluate (B220+cells); NTC: No Template Control D) Western blot of Control Spleens (Lanes 3-5) and splenic tumors from CD19-Cre mice (Lanes 6-8) shows lack of P53 expression in the tumors. BCL6 expression is seen in the tumors. Wild Type Brain (Lane 1) was used as positive control. Vinculin was used as the loading control; Original blots presented in Supplementary Figure 11

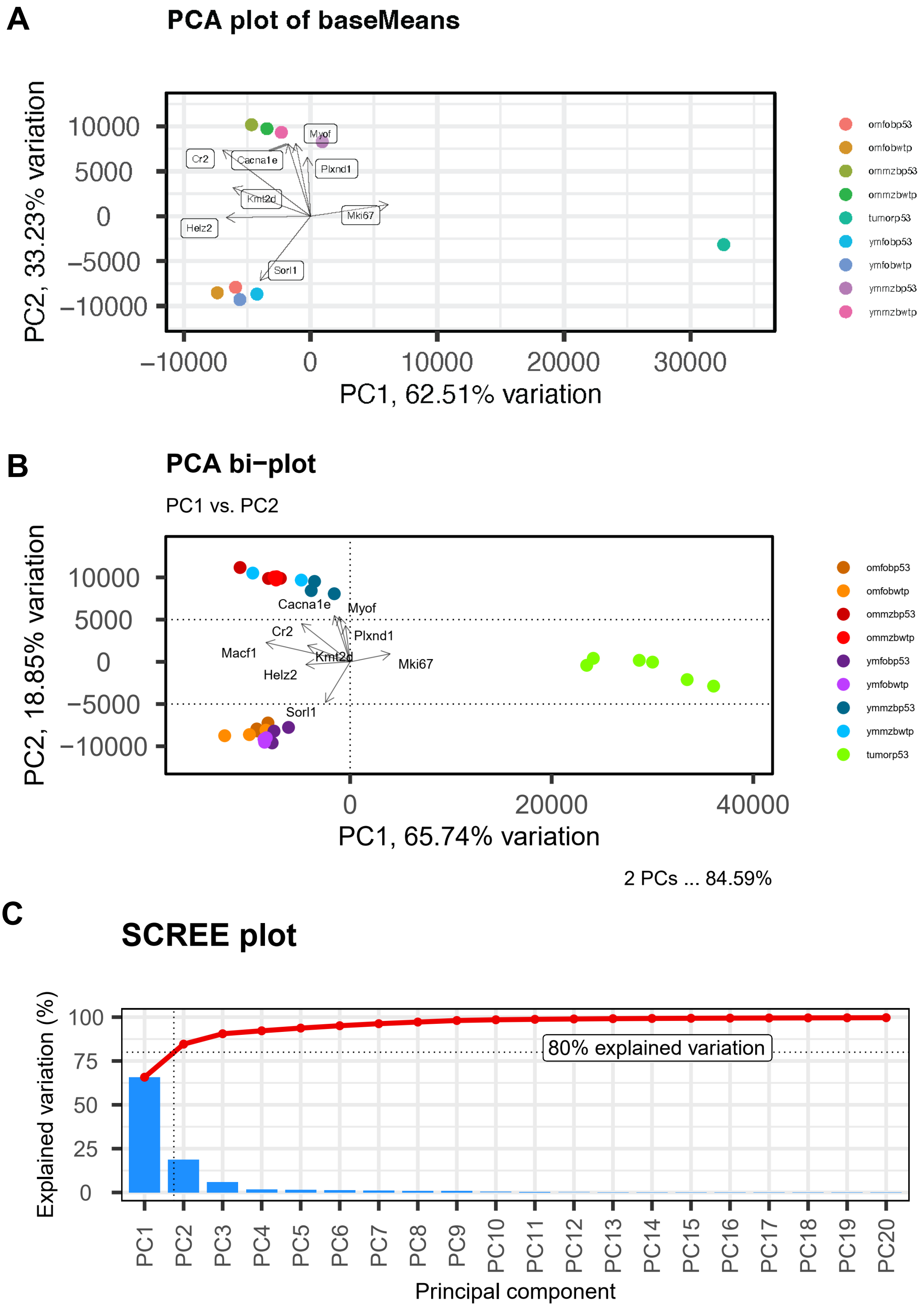

Supplementary Figure 6: PCA plot of A) Average of all samples B) Individual samples showing clustering of Follicular Zone cells/Marginal Zone cells and Tumors C) Scree plot highlighting the variation in different principal components;

Om: Old mouse (24 weeks), ym: Young mouse (12 weeks), fob: Follicular Zone cells, mz: Marginal Zone cells, wtp: Wild Type P53, p53: P53 knockout
