# Supplementary material for: Distinct oncogenic phenotypes in hematopoietic specific deletions of Trp53
Source: Sci Rep. 2023 May 9;13:7490. doi: 10.1038/s41598-023-33949-8 (PMC10169790; doi:10.1038/s41598-023-33949-8)
Supplement: Supplementary file 2 — Supplementary Information. [file 41598_2023_33949_MOESM2_ESM.pdf]

Supplementary Methods:

MACS sorting

Magnetic-activated cell sorting was performed using CD19+ microbeads and LS columns (Miltenyi Biotech) as described previously.

RNA isolation and RT-qPCR

RNA was isolated from mouse tissues using the Trizol method. This was (1000ng) reverse transcribed using iScript/qScript reagent (Quanta Biosciences). Real Time quantitative PCR was performed with the StepOne Plus Real-Time PCR System (Applied Biosystems) using PerfeCTa SYBR Green FastMix reagent (Quanta Biosciences) or TaqMan MicroRNA Assay (Life Technologies). The qPCR primer sequences used are listed in Supplementary Table 1.

Western Blot

Cells were lysed in RIPA buffer (Boston BioProducts) supplemented with Halt Protease and Phosphatase Inhibitor Cocktail (Thermo Scientific). Equal amounts of protein lysate (as quantified by using bicinchoninic acid protein assay, BCA (Thermo Scientific)) were electrophoresed on a 5–12% SDS–PAGE and electroblotted onto a nitrocellulose membrane. Antibodies used were anti-mouse NICD, β-tubulin, Total GSK-3 β, Phospho-GSK-3β, Numb, Mdm2 and β-Actin. Secondary HRP-conjugated antibodies were purchased from Santa Cruz Biotechnology. SuperSignal West Pico kit (Pierce) was used for enhanced chemiluminescence based detection.

Full-length western lots, where available, were provided to the journal during the review process. For some of the antibodies, we have cut the membranes so that multiple antibodies could be probed from a single run. Hence full-length blots are not available for some of the Western images.

RNA-sequencing and data analysis

Total RNA was extracted from tumors using Trizol combined with Qiagen miRNEasy mini kit with additional on column DNAse I digestion. Following isolation of RNA, cDNA libraries were built using the Illumina (San Diego, CA) TrueSeq RNA Sample Preparation kit V2 (RS-122-2001). An Agilent Bioanalyzer was used to determine RNA quality (RIN >8) prior to sequencing. RNA-Seq libraries were sequenced on an Illumina HiSeq 2000 (single-end 50bp). Raw sequence files were obtained using Illumina's proprietary software and will be made available at NCBI's Gene Expression Omnibus upon acceptance of the current manuscript.

The reads were mapped with Bowtie 2 (Langmead and Salzberg 2012) against the set of mouse repeat-masker elements in the mm10 assembly, as well as a constant poly-A sequence. Reads that were mapped to these elements were removed from further processing. The repeat-filtered full-length reads were mapped with STAR 2.5.3a (Dobin

et al. 2013), using the "--alignEndsType EndToEnd" option, to a single joint target consisting of the mm10 genome assembly. In cases of multiply-mapped reads, only the best mapping was retained. Mapping Statistics can be found in Supplementary Table 2 (Sheet 1).

Gene-by-gene coverage was extracted from the mm10 mappings that overlapped any potential exon for each gene in a gene model of mm10. The total coverage for each gene was divided by the total read length of each mapping to extract read counts as input to DESeq2 (Love, Huber, and Anders 2014). DESeq2 was run to compare all replicates of conditions as follows:

1. Old or young marginal zone wild type vs TP53 null
2. Old or young follicular samples wild type vs TP53 null
3. Young marginal zone samples from TP53 null mice to tumor samples
4. Young follicular samples from TP53 null mice to tumor samples

Complete sets of differentially expressed genes from all comparisons can be found in Supplementary Table 2 (Sheets 2-12).

Supplementary Table 1: List of qPCR primers used

Primers used	Sequence (5'-3')
Mapk13 F	CAACCTCCTCGCAGGACC
Mapk13 R	GTAGCTCCCAGGCAGTCTTG
Pik3cb F	TGATCGTTCACATCGCAGGG
Pik3cb R	CCTTCGGGAAAACCTGCACC
Sgk1 F	GAAGGCGGATCGGGATACAG
Sgk1 R	TGAGCATTTCTTGACCGGGA
Ccne1 F	GCTGCTAAGGAGGGTGCTAC
Ccne1 R	CAGCAACCTACAACACCCGA
Notch1 F	TGAGACTGCCAAAGTGTTGC
Notch1 R	GTGGGAGACAGAGTGGGTGT
Hes1 F	CGGCATTCCAAGCTAGAGAAGG
Hes1 R	GGTAGGTCATGGCGTTGATCTG
p21 F	ATTCCCTGCCTGGTTCCTTG
p21 R	AGTGGGGACCATTCCTGTCT
